# Artificial intelligence-driven design and optimization of mitochondria-targeted nanocarriers for chronic heart failure

**DOI:** 10.3389/fbioe.2026.1824349

**Published:** 2026-05-26

**Authors:** Zhengyi Zhang, Xiujuan Zhou, Tao Wei, Luanluan Meng, Kunlan Long

**Affiliations:** 1 School of Clinical Medicine, Chengdu University of Traditional Chinese Medicine, Chengdu, China; 2 Department of Critical Care Medicine, Hospital of Chengdu University of Traditional Chinese Medicine, Chengdu, China

**Keywords:** artificial intelligence, chronic heart failure, mitochondria, mitochondria-targeted, nanomedicine, review

## Abstract

Chronic heart failure (CHF) poses a substantial global public health challenge, yet contemporary therapeutic strategies remain predominantly confined to hemodynamic compensation without reversing the fundamental myocardial bioenergetic failure. Mitochondrial dysfunction represents the core pathological driver of disease progression; however, conventional pharmacological agents encounter substantial barriers in traversing systemic biological obstacles and the mitochondrial double-membrane system to achieve precise organelle targeting. This review systematically delineates advances in the convergence of artificial intelligence (AI) and nanotechnology for mitochondria-targeted therapy in CHF. We first dissect the pathological mechanisms encompassing mitochondrial dynamics dysregulation, energetic collapse, and quality control failure. We subsequently elucidate how AI-engineered nanocarriers hierarchically navigate multiple physiological barriers from administration routes to mitochondrial membranes, with particular emphasis on machine learning-optimized antioxidant nanoformulations, biomimetic membrane encapsulation technologies, and synthetic mitochondria constructs. This review aims to catalyze multi-omics-guided personalized mitochondria-targeted repair strategies, facilitating a paradigm shift from hemodynamic compensation to precision etiological treatment.

## 1 Introduction

Cardiovascular disease represents the leading cause of morbidity and mortality worldwide (Centers for Disease Control and Prevention and National Center for Health Statistics, 2026). Chronic heart failure (CHF) serves as the final common pathway for diverse structural heart diseases progressing to advanced stages, with its cardinal pathophysiological mechanism involving severe impairment of ventricular systolic and/or diastolic function. This precipitates reduced cardiac output, pulmonary and systemic congestion, and ultimately culminates in a vicious cycle of inadequate tissue perfusion and oxygen supply-demand mismatch (Kitai et al., 2025; Martin et al., 2025). Multiple etiologies, including acute myocardial infarction secondary to coronary artery disease, fulminant myocarditis, valvular heart disease, and inherited cardiomyopathies, converge upon CHF through myocardial remodeling and sustained neurohormonal activation (Savarese et al., 2023). Epidemiological evidence demonstrates that the 5-year survival rate following CHF diagnosis remains below 50%, approximating that of many malignancies. Moreover, with population aging and the escalating prevalence of metabolic syndrome, global disease burden is projected to increase by 46% from current estimates by 2030 (Khan et al., 2024). Characterized by high prevalence, frequent hospital readmissions, and substantial mortality, CHF imposes considerable healthcare resource utilization and socioeconomic burdens, constituting one of the most formidable public health challenges of the 21st century (Osenenko et al., 2022; GBD, 2023 Causes of Death Collaborators, 2025).

As summarized in Table 1, prior to the Guideline-Directed Medical Therapy (GDMT) era, heart failure management primarily relied on the classic “positive inotropic-diuretic-vasodilator” strategy, focusing on symptomatic hemodynamic compensation while failing to address underlying neurohormonal hyperactivation or myocardial bioenergetic remodeling. As an evidence-based, mechanism-driven therapeutic paradigm, GDMT advocates a foundational quadruple regimen comprising beta-blockers, sodium-glucose cotransporter-2 inhibitors, renin-angiotensin-aldosterone system inhibitors/angiotensin receptor-neprilysin inhibitors, and mineralocorticoid receptor antagonists. Through systematic antagonism of maladaptive neurohumoral responses, reduction of cardiac preload and afterload, and optimization of myocardial energetic metabolism (Maddox et al., 2024; Chinese Society of Cardiology et al., 2024; Savarese et al., 2024), this approach has become the cornerstone of therapy for heart failure with reduced ejection fraction. However, although GDMT effectively reduces the composite risk of cardiovascular death or heart failure hospitalization (Caleyachetty et al., 2015), its impact on hard endpoints, including all-cause mortality and rehospitalization rates, remains limited (Yancy et al., 2017), with therapeutic benefits progressively attenuating as left ventricular ejection fraction increases (Lassale et al., 2022). Notably, a large-scale cohort study demonstrated that merely 6.2% of patients achieved optimal GDMT dosing within 1 year of diagnosis (Sumarsono et al., 2024).

**TABLE 1:** Comparison between conventional therapies and GDMT.

Characteristics	Pre-GDMT Era	Guideline-directed medical therapy (GDMT)
Pharmacotherapy	Loop diuretics, digoxin, vasodilators	β-blockers, SGLT2 inhibitors, RAAS inhibitors/ARNI, MRAs
Therapeutic goal	Symptomatic hemodynamic compensation	Mechanism-driven neurohormonal antagonism and metabolic optimization
Mechanism of action	Reduction of cardiac preload/afterload; positive inotropy	Systemic inhibition of maladaptive sympathetic-RAAS activation; optimization of myocardial energetics
Impact on bioenergetics	No direct intervention	Partial metabolic remodeling
Key limitations	Failure to reduce mortality or interrupt adverse cardiac remodeling	Minimal impact on all-cause mortality and rehospitalization; progressively attenuated benefits with increasing LVEF; low real-world dosing optimization rates

Abbreviations: ARNI, angiotensin receptor-neprilysin inhibitor; GDMT, guideline-directed medical therapy; LVEF, left ventricular ejection fraction; MRAs, mineralocorticoid receptor antagonists; RAAS, renin-angiotensin-aldosterone system; SGLT2i, sodium-glucose cotransporter-2 inhibitor.

Non-pharmacological strategies, including cardiac resynchronization therapy implantable cardioverter-defibrillators, cardiac contractility modulation, and heart transplantation, provide incremental benefits in selected populations, yet their widespread adoption remains hampered by stringent eligibility criteria, donor organ scarcity, procedural invasiveness, and substantial economic burden. Therefore, elucidating the molecular underpinnings of progressive cardiomyocyte degeneration and bioenergetic collapse is imperative, with a strategic focus on etiological interventions capable of reversing myocardial injury—an approach that holds substantial promise for transcending current therapeutic limitations in heart failure.

Mitochondria have emerged as a promising therapeutic target, with accumulating evidence causally implicating mitochondrial alterations and oxidative stress in the development and progression of CHF (Hinton et al., 2024; Liu et al., 2022). As subcellular organelles pivotal to energy production, calcium signaling, and programmed cell death (Zhang et al., 2023a; Rizzuto et al., 2012), mitochondria not only govern oxidative phosphorylation-driven adenosine triphosphate (ATP) synthesis but also modulate excitation-contraction coupling through calcium sequestration and release, while maintaining reactive oxygen species (ROS) homeostasis and regulating cell death pathways (Monzel et al., 2023). Within cardiomyocytes, the dynamic distribution and intercellular transfer of mitochondria ensure efficient energy transmission and utilization, particularly during systolic contraction and diastolic relaxation (He et al., 2025). However, conventional small-molecule drugs, including metoprolol, bisoprolol, enalapril, valsartan, spironolactone, dapagliflozin, and others—as well as biologics such as recombinant natriuretic peptides, which constitute the cornerstone of chronic heart failure pharmacotherapy, universally lack subcellular targeting specificity. This limitation reflects a cross-disease bottleneck rather than a cardiac-specific defect: as demonstrated in oncology, the double-membrane architecture of mitochondria fundamentally prevents these agents from achieving therapeutic concentrations within the organelle (Biasutto et al., 2010). Although these conventional therapeutics alleviate congestion and improve hemodynamic compensation, they do not directly target the core pathological mechanisms underlying cardiac remodeling—specifically mitochondrial dysfunction, oxidative stress, and progressive bioenergetic failure (MacDonald et al., 2023). Compounded by systemic off-target toxicity, this failure of subcellular enrichment precludes effective clinical translation toward etiological repair, thereby necessitating the development of mitochondria-targeted nanocarriers. Consequently, the development of precision drug delivery systems capable of traversing dual biological barriers to achieve mitochondrial enrichment at the subcellular level has become a forefront research priority in precision therapeutics for heart failure (Hinton et al., 2024; Ji et al., 2025; Parker et al., 2025).

**FIGURE 1:**
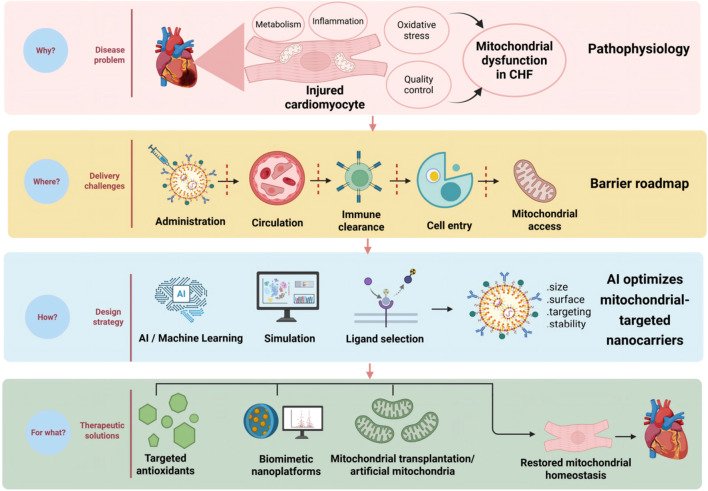
Summary Figure.

The convergence of nanomedicine and artificial intelligence (AI) is catalyzing a paradigm shift in CHF therapeutics, transitioning the field from proof-of-concept validation toward clinical translation (Figueiró Longo et al., 2023). At the subcellular theranostic frontier, mitochondria-localized deoxyribonucleic acid(DNA) nanosensors enable *in situ*, real-time detection and imaging of critical biomolecules governing mitochondrial homeostasis, spanning mitochondrial DNA (mtDNA) mutations, mitochondria-specific microRNAs, redox-sensitive enzymes, ROS, and calcium ions (Yu et al., 2024; Mazumdar et al., 2025). Concurrently, mitochondria-affine ligands facilitate subcellular-precision delivery across the double-membrane barrier, culminating in matrix enrichment (Wang et al., 2025). Through AI-driven mining of high-throughput biomedical datasets, novel drug nanocarriers are being identified and nanoscale properties optimized for distinct therapeutic contexts, ultimately enhancing nanomaterial safety and efficacy to enable precision matching of drug-carrier-patient triads (Mazumdar et al., 2025) (Figure 1).

**FIGURE 2:**
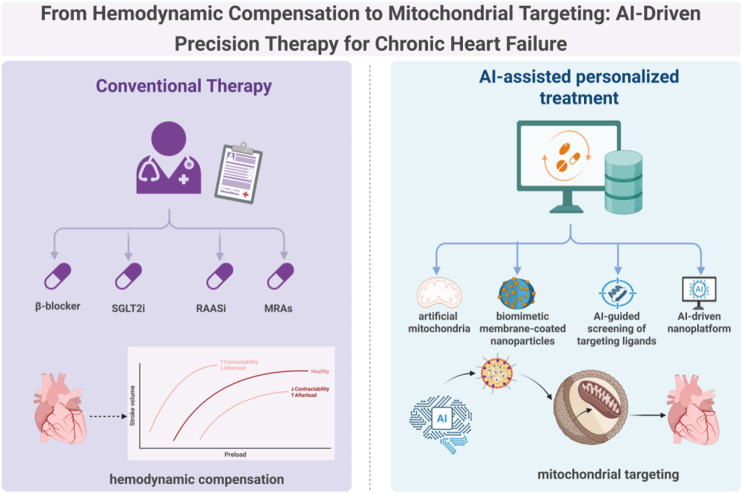
GDMT Therapy vs. AI-Assisted Personalized Treatment.

In this Review, we systematically delineate the therapeutic landscape at the convergence of AI and nanotechnology for mitochondria-targeted interventions in CHF. We first dissect the core pathological mechanisms underlying mitochondrial dysfunction during CHF progression. We subsequently explore AI-driven rational design strategies for nanocarriers, encompassing targeted ligand optimization, intelligent prediction of drug release kinetics, and generation of patient-specific dosing regimens, to achieve precision delivery traversing multiple biological barriers. Concurrently, we synthesize the translational potential of emerging therapeutic modalities, including mitochondria-targeted antioxidants, nanocarriers for mtDNA repair enzymes, and mitochondrial transplantation microcarriers. Finally, we critically evaluate prospective solutions to key translational challenges, including biosafety assessment, scalable manufacturing processes, and integrative Chinese and Western medicine evaluation frameworks. Our objective is to provide a theoretical foundation for developing precision therapeutic strategies capable of reversing myocardial bioenergetic failure, driving a paradigm shift from hemodynamic management toward etiological repair in heart failure, and ultimately improving clinical outcomes for patients (Figure 2).

## 2 Literature search strategy

This study systematically searched the Web of Science, PubMed, ScienceDirect, and China National Knowledge Infrastructure databases, with the search timeframe spanning from database inception to 1 February 2026. The search strategy employed a combination of Medical Subject Headings terms and free text words, focusing on three core concepts: “mitochondrial dysfunction and targeted interventions,” “nanotechnology and nanomedicine,” and “artificial intelligence and machine learning,” combined with terms related to chronic heart failure. Boolean operators were used to combine the search terms. The inclusion criteria were as follows: (a) clinical studies; (b) mechanistic studies; and (c) comprehensive reviews and systematic evaluations. Exclusion criteria included: (a) mini-reviews, commentaries, or editorials; (b) duplicate entries; and (c) non-Chinese or non-English literature where full texts were unavailable. Two researchers independently conducted the initial screening of titles and abstracts, followed by full-text assessment to determine final inclusion. Discrepancies were resolved through discussion or consultation with a third researcher to ensure study reliability. This review focuses on studies related to mitochondria-targeted nanotherapeutics and AI-assisted design strategies for cardiovascular diseases.

## 3 Mitochondrial pathophysiology in heart failure

### 3.1 Dysregulation of mitochondrial dynamics and morphological remodeling

Mitochondrial dynamics represent a core structural plasticity mechanism enabling cardiomyocytes to adapt to bioenergetic demands while maintaining oxidative phosphorylation efficiency. Through precise coupling of fusion and fission processes, these dynamics ensure dynamic equilibrium between network morphology and organelle function (Hinton et al., 2024). During CHF pathogenesis, this equilibrium shifts toward excessive fragmentation, characterized by significant upregulation of dynamin-related protein 1 (DRP1) concomitant with downregulation of mitofusin-2 (MFN2) and optic atrophy 1 (OPA1), constituting a pathological remodeling of the fusion-to-fission ratio (Sabbah et al., 2018; Hu et al., 2019). Such dysregulation not only induces geometric fragmentation of the mitochondrial reticulum but also compromises cristae ultrastructural integrity, thereby directly impairing ATP synthase efficiency and ATP synthesis (Caron and Bertolin, 2024).

DRP1 serves as the principal mediator of mitochondrial fission, with its enzymatic activity subject to allosteric regulation via phosphorylation cascades (Tong et al., 2020; Qi et al., 2019). Serine 637 located within the Guanosine triphosphatase (GTPase) regulatory domain functions as a critical “molecular rheostat”: phosphorylation mediated by cAMP-dependent protein kinase (PKA) inhibits DRP1 GTPase activity, blocking mitochondrial outer membrane constriction and promoting network fusion, thereby exerting cardioprotective effects (Sharp et al., 2014); conversely, calcineurin-mediated dephosphorylation relieves this inhibition, triggering DRP1 recruitment to the outer membrane and subsequent oligomerization, culminating in mitochondrial fragmentation and contractile dysfunction. Notably, during heart failure, cytosolic Ca^2+^ overload and sympathetic-adrenergic hyperactivation collectively constitute signals for calcineurin constitutive activation, maintaining DRP1 in a constitutively active state that accelerates cardiomyocyte apoptosis and metabolic remodeling (Ahola et al., 2022). Concurrently, OPA1 operates as a key effector for inner mitochondrial membrane fusion and cristae maintenance, with its functionality dependent on the balance between long isoform and short isoform. Within the heart failure microenvironment, oxidative stress and calcium dyshomeostasis activate inner membrane-associated metalloproteases, inducing aberrant OPA1 cleavage and depletion of L-OPA1. Consequently, this triggers cristae disorganization, disassembly of respiratory chain supercomplexes, and impaired autophagic flux, ultimately driving the execution of cell death programs (Wai et al., 2015).

Beyond these core regulatory axes, mitochondria may engage in compensatory hyperfusion as an adaptive survival strategy under stress. This process depends on the coordinated expression of stomatin-like protein 2, mitofusin-1, and long isoform optic atrophy 1 (L-OPA1) (Tondera et al., 2009), constituting a DRP1-independent regulatory axis that transiently preserves mitochondrial membrane potential and mtDNA stability. However, when compensatory fusion proves insufficient to reverse persistent bioenergetic crisis, this protective mechanism transforms into pathological metabolic rigidity, thereby promoting cardiomyocyte hypertrophy, interstitial fibrosis, and progression to heart failure (Haileselassie et al., 2019; Marín-García and Akhmedov, 2016; Beasley et al., 2021). Thus, dysregulated mitochondrial dynamics not merely manifests as a fragmented phenotype but represents a critical transition point from compensatory remodeling to decompensated cardiac deterioration.

### 3.2 Mitochondrial bioenergetic remodeling and oxidative stress

The inability to generate and transmit energy is recognized as a principal mechanism linking mitochondrial dysfunction to contractile failure (Zhou and Tian, 2018). Mitochondrial oxidative phosphorylation (OXPHOS) constitutes the predominant source of ATP synthesis in the myocardium (Ho et al., 2019; Diakos et al., 2016). Given the absence of significant ATP reserves in the heart, sustained contraction depends on immediate rephosphorylation of adenosine diphosphate (ADP) by mitochondria (Monzel et al., 2023); consequently, interruption of oxidative metabolism precipitates bioenergetic crisis within seconds. CHF represents a progressive bioenergetic failure (Lopaschuk et al., 2021; Tuomainen and Tavi, 2017; Azevedo et al., 2013), characterized not by mere depletion of total ATP pools, but rather by a mismatch between energy production efficiency and cardiac workload demands, alongside progressive decline in bioenergetic reserve capacity as reflected by the phosphocreatine (PCr)/ATP ratio (Mahmod et al., 2018; Hundertmark et al., 2023).

Mitochondrial dysfunction in CHF initially manifests as disruption of membrane lipid microenvironment. Ischemia and oxidative stress promote cardiolipin depletion from the inner mitochondrial membrane, compromising lipid anchoring of electron transport chain complexes (Lesnefsky et al., 2004) and subsequently triggering disassembly of respiratory chain supercomplexes (Rosca and Hoppel, 2010). Such structural fragmentation not only impairs NADH-cytochrome c reductase activity but also exacerbates electron leakage at Complexes I and III, culminating in superoxide burst (He et al., 2025; Sun et al., 2024; Giordano, 2005).

Progression of heart failure is accompanied by an obligate metabolic phenotype switch from fatty acid oxidation toward glycolysis (Stanley et al., 2005; Fukushima et al., 2015). Although glycolytic flux increases compensatorily to offset reduced mitochondrial ATP generation (Sun et al., 2024), the ATP yield per glucose molecule remains below 10% of that achieved through oxidative phosphorylation. This metabolic inflexibility (Brown et al., 2017) extends beyond the glucose-fatty acid shift to encompass impaired branched-chain amino acid catabolism (Hahn et al., 2023; Murashige et al., 2022), resulting in accumulation of toxic metabolites within the myocardium (Sun et al., 2016) that directly inhibit pyruvate dehydrogenase complex activity. Such inhibition further restricts glucose oxidation while inducing mitochondrial oxidative stress. Notably, aberrant epigenetic regulation involving DNA methyltransferases 3A and 3B also contributes to this pathological process, exacerbating contractile dysfunction through damage to mitochondrial cristae architecture and perturbation of glucose metabolism (Madsen et al., 2021; Madsen et al., 2020; Gilsbach et al., 2018).

Mitochondrial dysfunction encompasses epigenetic modifications of mtDNA, wherein faithful transcription is essential for maintaining organelle function and consequently preserving cellular bioenergetic homeostasis (Basu et al., 2020). In models of pressure overload–or ischemia-induced heart failure, p53-driven upregulation of methyltransferase-like 4 catalyzes elevated N6-methyladenine modification within mitochondrial promoter regions. This “fetal-like” epigenetic mark impedes recruitment of mitochondrial transcription factor A to mtDNA, resulting in transcriptional stasis of mitochondrial-encoded respiratory chain subunits (Zhang et al., 2024). Given that mtDNA encodes 13 core subunits of the OXPHOS apparatus, such repression directly compromises respiratory chain complex biogenesis, establishing an epigenetic-transcriptional-bioenergetic cascade of failure.

Mitochondria function as buffering reservoirs for cytosolic Ca^2+^, modulating OXPHOS rates via Ca^2+^-activated dehydrogenases to match contractile demands. In heart failure, impaired sarcoplasmic reticulum Ca^2+^ reuptake coupled with increased ryanodine receptor leakage results in delayed cytosolic Ca^2+^ clearance during diastole, chronically exposing mitochondria to elevated Ca^2+^ levels (Santulli et al., 2015; O-Uchi et al., 2014). Mitochondrial Ca^2+^ overload not only triggers opening of the mitochondrial permeability transition pore, culminating in cell death, but also compromises redox homeostasis through dissipation of mitochondrial membrane potential and depletion of reduced nicotinamide adenine dinucleotide phosphate pools, thereby rendering energy supply unable to respond to acute contractile demands (Maack et al., 2006).

Mitochondria-dependent cell death cascades mediated by ROS have been repeatedly observed following chronic neurohumoral activation, resulting in global deterioration of mitochondrial function (Izem-Meziane et al., 2012; Léger et al., 2011). Furthermore, ROS damages proteins and lipids, triggers cell death pathways, and induces synchronous collapse of the cellular energy network (Zorov et al., 2006; Aon et al., 2006). Lacking histone protection and possessing limited repair machinery, mtDNA is particularly vulnerable to ROS-induced mutations and deletions, which further diminish expression of respiratory chain subunits, thereby establishing a self-amplifying “ROS damage-dysfunction-more ROS” vicious cycle. This process is accompanied by downregulation of the peroxisome proliferator-activated receptor gamma coactivator 1-alpha/estrogen-related receptor alpha (PGC-1α/ERRα) pathway, impeding mitochondrial biogenesis and ultimately driving the transition of cardiomyocytes from compensatory hypertrophy to decompensated failure (Karamanlidis et al., 2010; Russell et al., 2004; Arany et al., 2005).

A decreased PCr/ATP ratio serves as a cardinal indicator of compromised bioenergetic reserve (Weiss et al., 2005). This reduction predominantly reflects declining PCr levels, as ATP concentrations remain relatively stable in the failing heart, persisting even into the terminal stage (Smith et al., 2006). Non-invasive phosphorus-31 (^31^P) magnetic resonance spectroscopy studies have demonstrated significant reductions in myocardial PCr/ATP ratios across the heart failure spectrum, encompassing both heart failure with reduced ejection fraction and heart failure with preserved ejection fraction (Phan et al., 2009). Crucially, this reduction is not merely a static biochemical metric; rather, it portends an impaired capacity to sustain ATP homeostasis under physiological stress, signifying that bioenergetic reserves have fallen below a critical threshold and endogenous mitochondrial compensatory mechanisms have become exhausted. This bioenergetic crisis does not represent a passive pathological consequence; instead, it functions as a proximal driver of heart failure progression, actively perpetuating a vicious cycle of cardiac remodeling (Zhou and Tian, 2018).

### 3.3 Collapse of mitochondrial quality control

Mitochondrial quality control constitutes a hierarchical defense system essential for cardiomyocyte homeostasis maintenance, comprising multi-layered mechanisms including chaperone-protease repair, the mitochondrial unfolded protein response (UPR^mt^), selective autophagy, and mitochondrial biogenesis. Given the central role of mitochondria in cardiac bioenergetics, compromise of this integrated surveillance network inevitably results in accumulation of defective mitochondria, cell loss, and ventricular remodeling, ultimately driving progression to heart failure.

The Dichotomous Role of the Unfolded Protein Response. Accumulation of misfolded proteins within the mitochondrial matrix activates the UPR^mt^ (Smyrnias et al., 2019; Houtkooper et al., 2013), leading to upregulation of chaperones and proteases via Activating Transcription Factor 5 (ATF5) and C/EBP Homologous Protein (CHOP), thereby transiently preserving proteostasis (Fiorese et al., 2016; Guo et al., 2020). During CHF progression, however, this defensive system undergoes a “hierarchical collapse”: early-stage persistent UPR^mt^ activation accompanied by a shift of ATF5/CHOP signaling from cytoprotective to pro-apoptotic; mid-stage impairment of PINK1/Parkin-mediated mitophagic flux and subsequent accumulation of p62/SQSTM1; and late-stage compensatory upregulation of alternative clearance pathways driven by lysosomal failure, which ultimately proves insufficient to reverse mitochondrial damage (Ravindran and Gustafsson, 2025).

Mitophagy denotes the process whereby cells selectively degrade senescent, damaged, or dysfunctional mitochondria via autophagy, playing a pivotal regulatory role in maintaining mitochondrial quality (Lemasters, 2005). The PINK1-Parkin pathway constitutes the canonical mechanism governing this process (Yamamoto et al., 2023). As mitochondrial function declines, an isoform switch from AMP-activated protein kinase (AMPK)α2 to AMPKα1 occurs. Upon phenylephrine stimulation, AMPKα2 phosphorylates PINK1, thereby facilitating PINK1/Parkin-mediated mitophagy, enhancing clearance of impaired mitochondria, and consequently ameliorating organelle function (Wang B. et al., 2018). However, ATP depletion in advanced CHF inhibits ubiquitin-proteasome system activity, resulting in accumulation of Parkin substrates and ubiquitinated proteins, and thereby creating a pathological stalemate of blocked mitophagic flux (Lazarou et al., 2015; Stolz et al., 2014). Uncleared fragmented mitochondria persistently generate ROS, reciprocally damaging adjacent healthy mitochondria and perpetuating the cycle of mitochondrial injury.

**FIGURE 3:**
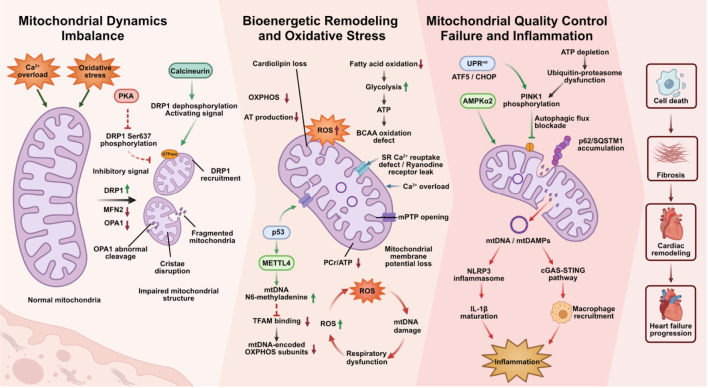
Pathophysiological mechanisms of mitochondrial dysfunction in chronic heart failure.

The ultimate manifestation of quality control failure is characterized by the release of mitochondrial constituents and the consequent initiation of systemic inflammation (Russell et al., 2004). Damaged mitochondria discharge mtDNA, which activates innate immunity via the NLRP3 inflammasome (Nakahira et al., 2011) and the cyclic GMP-AMP synthase–stimulator of interferon genes pathway (Riley and Tait, 2020), promoting interleukin-1β maturation. Simultaneously, when PINK1/Parkin-mediated mitophagy is impaired, diminished Beclin 1-dependent clearance results in persistent accumulation of mitochondrial damage-associated molecular patterns (Sun et al., 2018), further driving macrophage recruitment. Notably, upon lysosomal failure, cardiomyocytes expel damaged mitochondria via extracellular vesicles (Liang et al., 2023), transiently alleviating intracellular mitochondrial load; however, subsequent engulfment by macrophages feedback-activates inflammatory responses (Nicolás-Ávila et al., 2020). Ultimately, this establishes an irreversible vicious cycle of “mitochondrial damage–inflammation–cardiac remodeling” (Figure 3).

## 4 Traversing barriers: from routes of administration to subcellular delivery

In CHF therapeutics, clinical translational success critically depends upon the capacity of delivery systems to surmount the hierarchical anatomical and physiological barriers spanning from the site of administration to subcellular targets. As a frontier in current research, mitochondria-targeted nanomedicines hold promise for transcending traditional limitations and enabling emerging therapeutic paradigms—including modulation of mitophagy, mitochondrial quality control, calcium homeostasis regulation, and precise delivery of genetic materials (Tabish and Lygate, 2023; Bonora et al., 2019). However, a stringent cascade of obstacles—encompassing systemic barriers (route of administration selection), circulatory barriers (mononuclear phagocyte system interception and hemorheological challenges), tissue barriers (cardiac microvascular endothelium), and organelle barriers (endosomal/lysosomal entrapment and the mitochondrial double membrane)—constitutes a formidable hierarchy impeding delivery efficiency (Lin et al., 2021). Only through rational engineering of nanomaterials to achieve hierarchical traversal and active recognition across these barriers can effective drug enrichment from systemic circulation to the mitochondrial matrix be realized, ultimately unlocking the therapeutic potential of subcellular precision intervention.

### 4.1 Selection of routes of administration

Traditional oral administration confronts formidable bioavailability challenges in the chronic management of CHF: the tight junction architecture of intestinal epithelium, electrostatic repulsion by the mucus layer, metabolic transformation by gut microbiota, and the intestinal capillary barrier collectively restrict transmembrane drug transport efficiency. Furthermore, the acidic environment of the gastrointestinal tract, enzymatic degradation, and hepatic first-pass effect render therapeutic agents incapable of effective extravasation into myocardial target tissues (Anselmo et al., 2019).

Pulmonary delivery strategies exploit the unique anatomical and physiological features of the lungs to offer a non-invasive alternative for cardiac-targeted drug administration. The substantial surface area available for gas exchange, favorable epithelial permeability, and extensive capillary network enable nanoscale particles deposited within alveolar spaces to rapidly and efficiently traverse the air-blood barrier into systemic circulation (Patton and Byron, 2007). This distinct physiological advantage permits therapeutics to bypass hepatic first-pass metabolism and achieve direct extravasation into diseased myocardium. Preclinical investigations have demonstrated that nanoparticles encapsulating therapeutic peptides can successfully reach pathological cardiac tissue via this route (Miragoli et al., 2018; Modica et al., 2021).

Inhalable cardiac-targeted nanosystems demonstrate distinctive translational potential. Biodegradable calcium phosphate (CaP) nanoparticles have been harnessed as carriers for therapeutic peptides or microRNAs in heart failure therapeutics (Modica et al., 2021). Chen S et al. (Chen et al., 2020) and Weng H et al. (Weng et al., 2024) engineered biodegradable CaP nanoparticles functionalized with cardiac targeting peptide (CTP), designated TP-10@CaP-CTP, which encapsulate the selective phosphodiesterase 10A inhibitor TP-10. This nanoformulation enables precise modulation of cAMP/cGMP levels within cardiomyocytes and cardiac fibroblasts: via the cAMP/AMPK signaling pathway to suppress pathological cardiomyocyte hypertrophy, and through the cGMP/protein kinase G (PKG) pathway to inhibit activation, proliferation, migration of cardiac fibroblasts and extracellular matrix synthesis, thereby synergistically reversing cardiac hypertrophy and fibrotic remodeling. CTP modification confers active targeting capabilities toward diseased myocardium, which, in concert with the pulmonary delivery route, significantly promotes local cardiac enrichment of TP-10, improving cardiac function in pressure overload-induced heart failure models while circumventing systemic exposure-associated adverse effects. Weng H further demonstrated that the efficient delivery achieved through inhalation combined with CTP decoration enables targeting of pathological myocardium, enhancing local accumulation of the nanomedicine and thereby achieving superior therapeutic efficacy of TP-10 while preventing adverse pulmonary reactions (Weng et al., 2024). Additionally, a 7-day daily inhalation toxicity study conducted by Alogna et al. confirmed that inhalable dry powder CaP nanoparticles exhibit favorable long-term biosafety; notably, 2 weeks of inhalation significantly ameliorated CHF-associated pulmonary congestion and injury, establishing a safety foundation for clinical translation of inhalable nanomedicines (Alogna et al., 2024).

### 4.2 Biological barriers

#### 4.2.1 Activation of the mononuclear phagocyte system

The Mononuclear Phagocyte System (MPS) constitutes the primary biological barrier encountered by nanomedicines during transport from systemic circulation to myocardial tissue. Upon exposure to physiological environments, the high surface free energy of nanoparticles rapidly induces nonspecific adsorption of plasma proteins, forming a “protein corona” (Lazarovits et al., 2015). This transformation renders particles highly susceptible to recognition by pattern recognition receptors on Kupffer cells in the liver and marginal zone macrophages in the spleen, leading to rapid clearance from circulation via opsonin-mediated phagocytosis (George et al., 2022).

To evade MPS-mediated clearance, current strategies focus on surface chemical camouflage and phagocytic saturation interventions (Sheng et al., 2009; Proffitt et al., 1983). Polyethylene glycol (PEG) ylation represents the classical approach, wherein PEG chains covalently conjugated to the nanocarrier surface form a hydrophilic brush layer generating steric hindrance effects, effectively suppressing plasma protein adsorption. Notably, PEG2000-modified liposomes significantly attenuate complement consumption and prolong systemic circulation half-life, providing a temporal window for cardiac targeting and accumulation (Sheng et al., 2009; Kanamala et al., 2018; Mustafa et al., 2017). Biomimetic cell membrane coating technologies offer innovative biologically inspired strategies for MPS evasion. These constructs integrate synthetic nanoparticle cores with biologically derived membranes (e.g., erythrocyte membranes, platelet membranes, or macrophage membranes), preserving the multifunctional surface characteristics of native cellular membranes. This architectural design not only endows nanoparticles with the capacity to interact with host biological interfaces but, more critically, enables evasion of pattern recognition receptor recognition by Kupffer cells and splenic macrophages through inheritance of “self” recognition signals from the membrane surface, thereby markedly inhibiting opsonization and subsequent phagocytic clearance (Lu et al., 2023; Li et al., 2022; Cui et al., 2024). MPS saturation and uptake blockade strategies involve pretreatment to induce macrophage phagocytic saturation or transient inhibition of primary uptake functions, reducing hepatic and splenic sequestration of carriers and redirecting a greater fraction of the drug payload toward target organs such as the heart (Saunders et al., 2020; Sakurai et al., 2002).

From the perspective of strategic evolution, MPS blockade approaches have progressively transitioned from initial immunological research tools to pivotal enabling technologies for auxiliary gene and drug delivery, now constituting a central strategy for prolonging nanomedicine systemic circulation residence time and enhancing target tissue accumulation efficiency. *In vitro* experiments demonstrate that such biomimetic systems not only specifically adhere to cardiac collagen fibers via platelet membrane components but also markedly attenuate macrophage phagocytosis through immune-evasive molecules present on erythrocyte membranes. *In vivo*, in heart failure models induced by transverse aortic constriction and myocardial infarction, prolonged intravenous administration resulted in significant improvements in cardiac function, reduced myocardial fibrosis, and effectively attenuated off-target toxicity of JQ1 to extra-cardiac organs such as the liver and kidneys. These findings robustly validate the dual advantages of this strategy in evading MPS clearance and achieving cardiac-targeted therapeutics (Li Y. et al., 2024).

#### 4.2.2 Barrier 2: hemorheology

Having evaded MPS-mediated clearance, nanocarriers immediately confront the hemorheological barrier—the second physical obstacle restricting their delivery to myocardial tissue. Studies indicate that nanoparticle adhesion rates correlate with endothelial wall shear stress and the geometric complexity of the circulatory system (Lin et al., 2021; Hossain et al., 2013; Tan et al., 2011). Within the rapidly pulsatile bloodstream, erythrocytes undergo axial migration due to hydrodynamic interactions, forming a dense core that radially displaces nanoparticles toward the cell-free plasma layer adjacent to the vascular wall. This “margination” process constitutes the prerequisite for particle-endothelial contact. However, complex interactions exist between nanocarrier physicochemical properties and hemodynamic shear forces: submicron particles experience intense Brownian motion that prevents effective margination against fluid shear, whereas oversized particles are readily repelled from the wall by erythrocyte collisions under shear gradients, significantly diminishing vascular adhesion probability (Longmire et al., 2008). Furthermore, the elevated viscosity and non-Newtonian rheological characteristics of blood further restrict particle transverse diffusion, such that under physiological flow conditions, less than 1% of the injected dose successfully overcomes hemorheological barriers to achieve effective adhesion to cardiac microvascular endothelium.

To surmount these hemorheological constraints, rational nanocarrier engineering necessitates synergistic optimization across three dimensions: size, morphology, and mechanical compliance. Size optimization: The 5–200 nm range represents the optimal window for evading renal filtration (<5 nm) and mechanical interception by hepatic sinusoids (>200 nm) (Longmire et al., 2008). Specifically, nanoparticles of 100–200 nm demonstrate superior performance in the enhanced permeability and retention effect within infarcted and ischemic myocardium, capable of avoiding rapid glomerular clearance while achieving effective retention in ischemic cardiac microvasculature (George et al., 2022; Kamal et al., 2025; Lundy et al., 2016). Morphological engineering: Non-spherical particles exhibit superior margination efficiency compared to spherical counterparts. Ellipsoidal particles, in particular, possess greater surface area for wall adhesion within the cell-free layer, and their slower rotational dynamics enable deceleration of rolling motion and stabilization of adhesion, thereby significantly enhancing vascular wall attachment efficiency under shear flow conditions (Müller et al., 2014). Mechanical flexibility: The deformability of carriers exerts a decisive influence on hemorheological behavior. Rigid particles are prone to mechanical entrapment within capillaries and rapid MPS clearance, whereas flexible nanogels can adapt to hemodynamic forces through compliant deformation, prolonging systemic circulation time while enhancing tissue interstitial penetration to create favorable conditions for transendothelial transport (Blanco et al., 2015).

#### 4.2.3 Barrier 3: myocardial microvascular endothelium and contractile extravasation

In contrast to the Enhanced Permeability and Retention effect characteristic of tumor neovasculature, normal cardiac microvascular endothelial cells establish a highly selective continuous barrier via tight junctions and adherens junctions, effectively restricting transendothelial transport of nanomedicines. Although regional ischemia in CHF upregulates vascular endothelial growth factor expression and increases endothelial permeability, the therapeutic window for effective EPR remains transient (Dvir et al., 2011). Pathological myocardial fibrosis, characterized by excessive extracellular matrix deposition, induces tissue stiffening and architectural disruption (Henderson et al., 2020; Herrera et al., 2018), further increasing interstitial density to create a paradoxical “leaky yet dense” microenvironment—wherein increased vascular permeability is counteracted by a dense fibrotic network that impedes nanoparticle penetration into deep myocardial tissue. Consequently, the development of active targeting strategies becomes imperative to breach the cardiac microvascular endothelial barrier and achieve effective drug accumulation within diseased myocardium.

Cardiac targeting peptide (CTP) has been extensively validated as an effective ligand for targeted delivery in ischemic heart disease (Sun et al., 2023; Guan et al., 2022). While precise molecular targets remain incompletely characterized, existing evidence demonstrates that CTP-functionalized nanocarriers significantly enhance therapeutic accumulation within pathological myocardium (Wang X. et al., 2018; Kanki et al., 2011), potentially mediated by high-affinity binding to stress fiber-associated proteins on cardiomyocyte surfaces or to ischemia-induced membrane receptors. Nevertheless, CTP modification confronts the risk of a “binding site barrier”—wherein excessively dense ligand-receptor interactions may result in nanoparticle retention at the vascular endothelial surface, paradoxically impeding further extravasation into tissue interstitium.

Biomimetic Membrane Coating for Matrix Targeting.To address the pronounced interstitial fibrosis characteristic of advanced CHF, multifunctional biomimetic nanoparticle drug delivery systems harnessing the fusion of platelet and erythrocyte membranes (designated:PM&EM NPs) exhibit a distinctive endothelial escape–matrix homing cascade targeting capability. Integrin αIIbβ3 and P-selectin expressed on the platelet membrane surface specifically recognize cardiac myofibroblasts and exposed collagen, conferring upon nanocarriers the capability for active migration toward fibrotic foci following endothelial traversal. Concurrently, erythrocyte membrane incorporation provides “self” recognition signals, significantly prolonging systemic circulation residence time and attenuating MPS clearance. The synergistic interplay between these components effectively circumvents drug sequestration barriers within the fibrotic microenvironment (Li Y. et al., 2024).

Contraction-Mediated Extravasation Enhancement.The periodic mechanical stress generated by the heart’s rhythmic systolic-diastolic motion drives a “pump-driven” active transport of nanoparticles from the vascular lumen into tissue interstitium. This mechanism is particularly pronounced in ultrasmall nanocarriers: following inhalation administration, biocompatible and biodegradable calcium phosphate nanoparticles (CaPs), upon traversing the alveolar-capillary barrier into systemic circulation, exhibit superior deformability conferred by their nanoscale dimensions. This enables compliance with hemodynamic forces and generates a “squeeze-and-release” effect at capillary constrictions. Synergized with cardiac cyclic mechanical stress, CaPs more readily overcome endothelial tight junctions, achieving effective penetration into the ischemic border zone and intracellular enrichment (Miragoli et al., 2018).

#### 4.2.4 Barrier 4: endosomal/lysosomal degradation and mitochondrial penetration

Following endocytic entry into cardiomyocytes (Canton and Battaglia, 2012), nanomedicines confront the final physiological barrier en route from extracellular space to subcellular targets—the endosomal/lysosomal degradation pathway. Nanoparticles are initially internalized via clathrin-mediated endocytosis or macropinocytosis, forming early endosomes that progressively mature into late endosomes and ultimately fuse with lysosomes enriched in over 60 species of hydrolytic enzymes (Bae et al., 2012; Wang F. et al., 2018). Within this acidic microenvironment, both nanocarriers and their cargo face the risk of complete degradation. Consequently, effective delivery systems must protect nucleic acids from endonuclease and exonuclease attack while facilitating endosomal escape to circumvent lysosomal entrapment (Witzigmann et al., 2020).

To circumvent lysosomal fate, membrane fusion mechanisms represent an efficient escape strategy. Distinct from the proton sponge effect, membrane fusion achieves cytoplasmic release through direct disruption of endosomal membrane integrity via lipid phase transition. Rationally designed membrane fusion strategies significantly enhance the multifunctional characteristics of targeted drug delivery systems (Zheng et al., 2025). The MITO-Porter—an octaarginine peptide (R8)-functionalized liposomal nanocarrier—can directly transport cargo into the mitochondrial matrix through membrane fusion, bypassing lysosomal degradation steps (Buchke et al., 2022). Additionally, CaPs exploit their biomineralization properties to dissolve within acidic endosomal environments, successfully traversing cardiomyocyte membranes and intracellularly releasing bioactive molecules *in vitro* without inducing cytotoxicity or interfering with cellular functional properties (Di Mauro et al., 2016), thereby providing an alternative escape pathway based on inorganic nanomaterials.

**FIGURE 4:**
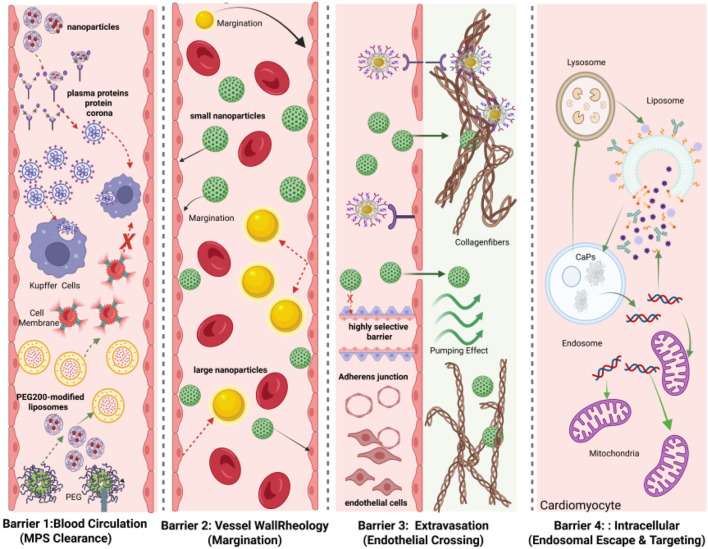
Traversing barriers: from routes of administration to subcellular delivery.

Lipid-Based Delivery Systems,AI-assisted design enables optimization of endosomal escape capabilities in ionizable lipid nanoparticles (LNPs) (Gil-Cabrerizo et al., 2024). Upon encountering acidic endosomal environments, charge reversal facilitates membrane fusion while simultaneously protecting mRNA from ribonuclease-mediated degradation (Soroudi et al., 2024). Following successful cytoplasmic release, positively charged mitochondria-targeting ligands exploit the negative membrane potential of the inner mitochondrial membrane to drive accumulation, thereby establishing a cascade delivery pathway from “endosomal escape” to “mitochondrial penetration” (Figure 4).

## 5 AI-driven nanocarrier design

Nanomedicine represents a paradigm-shifting approach with transformative potential for cardiovascular therapeutics. Although significant advances have been achieved in tissue targeting and payload stability, the path toward clinical translation remains fraught with challenges (Gao et al., 2026). AI technologies—encompassing machine learning, deep learning, neural networks, cognitive computing, and big data analytics (Yan et al., 2019)—facilitate mining of high-throughput biomedical data not only to identify potential drug interactions, predict therapeutic efficacy, and conduct high-throughput screening, but also to guide rational reconstruction and optimization of chemical structures for targeted delivery vehicles (Song et al., 2025).

### 5.1 Machine learning optimization of nanocarrier properties

AI algorithms analyze molecular structures, formulation components, and environmental factors to provide precise predictions for nanocarrier formulation design, ensuring optimal drug delivery and therapeutic outcomes (Li et al., 2012; Bannigan et al., 2023). In nanomedicine development, machine learning technologies have demonstrated the capability to autonomously extract signal features and detect weak biomarker signals, effectively addressing the high-dimensional data challenges posed by complex biological signals (Tittl et al., 2019).

Leveraging these capabilities—specifically autonomous feature extraction, sensitive biomarker detection, and high-dimensional biological data interpretation—machine learning exhibits distinct advantages in optimizing carrier designs for evading MPS recognition. By integrating multi-omics annotations with machine learning-driven pooled screening (Boehnke et al., 2022), algorithms can identify critical variables governing nano-bio interactions (Rao et al., 2024) and construct quantitative structure-activity relationships linking nanoparticle physicochemical parameters to vascular wall adhesion probability. This enables rational guidance for the design of surface PEGylation density and chain length; through optimization of surface chemistry, size, and payload release mechanisms, these algorithms minimize protein corona formation and prolong blood half-life.Furthermore, AI facilitates the creation of nanocarriers for targeted drug administration, reducing off-target exposure to healthy tissues and delivering therapeutic agents directly to diseased cells or tissues. This approach not only minimizes the requirement for experimental animal trials during drug formulation development but also shortens development timelines and enhances product quality (Bao et al., 2023), providing technical assurance for green and efficient development of nanomedicines for heart failure.

### 5.2 Molecular dynamics simulation of nano-bio interactions

Molecular dynamics (MD) simulation serves as a computational technique for replicating molecular behavior within specified force fields, providing atomistic-resolution tools for dissecting nano-bio interface interactions (Agha et al., 2023). Sarker P et al. utilized multiscale simulations to investigate protein corona formation on silver nanoparticle surfaces, identifying unique fingerprints of corona formation dynamics and peptide structural alterations, thereby guiding the design of hydrophilic nanoparticles adapted to complex biological environments (Sarker et al., 2022). For heart failure therapeutics, MD simulations enable investigation of intricate interactions among drugs, water molecules, and carriers within complex systems, predicting critical nanomaterial properties at the atomic level to provide a structural basis for breaching the cardiac microvascular endothelial barrier (Zhang et al., 2021). Furthermore, data mining and machine learning can be applied in nanoinformatics to predict nanoparticle cytotoxicity (Ahmadi et al., 2024; Banaye Yazdipour et al., 2023), assisting drug formulation processes through prediction and simulation of drug-carrier interactions to enhance encapsulation efficiency and controlled release kinetics (Ganju and Gupta, 2023). This strategy not only mitigates nanotoxicity risks but also establishes a structural biology foundation for breaching the cardiac microvascular endothelium and achieving efficient cardiac targeting through rational design of surface chemistry and carrier architecture.

### 5.3 Intelligent screening of targeting ligands

To address the bottleneck of endosomal/lysosomal escape, AI and machine learning tools provide intelligent solutions for enhancing intracellular stability and targeted release efficiency of nucleic acid therapeutics through rational design of RNA structures and LNP formulations (Castillo-Hair and Seelig, 2022; Rurik et al., 2022; Yang et al., 2022). Deep learning algorithms can decode thermodynamic principles governing RNA folding and establish structure-function relationships (Castillo-Hair and Seelig, 2022), while AI models based on computational linguistics frameworks systematically optimize codon usage preferences, secondary structure stability, and immunogenicity of mRNA, significantly accelerating the entire workflow from sequence design to functional prediction (Zhang et al., 2023b). More critically, machine learning establishes multidimensional mappings between molecular descriptors and intracellular behavior, enabling precise prediction of electrostatic interactions between LNPs and mRNA, encapsulation stability, and membrane fusion kinetics. This ensures structural integrity and controlled release of nucleic acid payloads during cellular membrane traversal and lysosomal evasion (Yang et al., 2022). Recent investigations have achieved breakthrough progress in integrating machine learning with high-throughput experimental screening, wherein computational pre-screening of LNP candidate libraries can shorten traditional experimental optimization cycles by several-fold, providing a scalable technical path for improving endosomal escape efficiency (Rurik et al., 2022). As AI-driven next-generation LNP carriers continue to evolve, their applications have expanded beyond conventional mRNA delivery toward precision delivery of CRISPR gene editing tools and development of intelligent nanorobots. These advances enhance therapeutic indices while reducing off-target toxicity, opening new technological paradigms for subcellular precision interventions such as mitochondrial gene therapy (Hasanzadeh et al., 2022).

Li et al. established a predictive lipid structure-function model utilizing PaDEL molecular descriptors through the integration of machine learning and high-throughput experimental screening (Li B. et al., 2024). Employing the XGBoost algorithm, the investigators trained models on *in vitro* transfection efficiency and endosomal escape metrics of 584 distinct LNPs, enabling prospective activity prediction across a large-scale virtual lipid library, with off-batch validation revealing performance significantly surpassing that of commercial benchmark formulations. Complementing this approach, AI-Guided Ionizable Lipid Engineering represents a parallel platform leveraging deep learning and combinatorial chemistry for the development of ionizable lipids tailored for mRNA delivery (Hasanzadeh et al., 2022), facilitating efficient lipid library design, synthesis, and comprehensive *in silico* screening. Such “computational design–experimental validation” paradigms not only substantially compress LNP optimization timelines but also enhance endosomal escape efficiency of nucleic acid therapeutics through precise modulation of lipid chemical architectures. Consequently, these strategies furnish scalable, intelligent screening platforms that accelerate clinical translation across diverse therapeutic modalities, encompassing CAR-T cell therapy, gene editing, and mRNA vaccines (Xu et al., 2024).

### 5.4 AI-driven personalized nanomedicine

The advent of the precision medicine era heralds a transformative shift from standardized “one-size-fits-all” therapeutic modalities toward individualized treatment paradigms (Mhatre et al., 2023). By integrating multi-omics datasets—encompassing genomics, proteomics, metabolomics—and clinical phenotypic parameters, AI enables the construction of multidimensional patient profiles, facilitating tailored engineering of nanocarriers (Mazumdar et al., 2025; Alshawwa et al., 2022). Such data-driven personalization optimizes not only the physicochemical attributes of carriers—including surface chemistry, geometric configurations, and release kinetics—but also leverages continuous learning from real-world evidence to dynamically adjust dosing regimens, accommodating the temporal evolution of individual pathophysiological characteristics.

In the realm of cardiovascular targeted delivery, AI-driven personalized nanomedicine exhibits distinctive advantages. Based on patient-specific vascular inflammatory profiles, endothelial barrier integrity, and myocardial metabolic status, algorithms can optimize surface functionalization density and targeting ligand selection of nanocarriers, achieving efficient recognition of diseased vascular endothelium and facilitating transendothelial transport (Omidian et al., 2023; Xitong and Xiaorong, 2016). Through precise modulation of carrier-biointerface interactions, such systems significantly enhance drug accumulation rates within lesion areas while reducing complications associated with off-target effects, thereby providing technical underpinning for precision intervention in heart failure patients.

Nevertheless, the translational gap between *in vitro* predictive models and *in vivo* physiological environments remains a bottleneck constraining nanomedicine development (Paunovska et al., 2018). Traditional *in vitro* assays fail to recapitulate complex variables such as tissue microarchitecture, hemodynamic forces, and immune system interactions, resulting in deviations in preclinical efficacy assessments (Gao et al., 2026). To address this limitation, the NanoMAP platform developed by Hickman et al. integrates high-throughput experimentation with machine learning algorithms to establish predictive models bridging *in vitro* screening and *in vivo* validation. The data-sharing paradigm advocated by this platform further fosters interdisciplinary collaboration within pharmaceutical sciences, accelerating the standardized development of next-generation nanomedicines (Hickman et al., 2023).

Ultimately, AI facilitates the creation of nanocarriers for targeted drug administration, minimizing exposure to healthy tissues while delivering therapeutic agents directly to diseased cells or tissues (Blanco-González et al., 2023; Su and Kang, 2020; Vidhya et al., 2023). Furthermore, nanorobots integrated with machine learning algorithms can parse molecular biomarker signals in real time, autonomously chart biological pathway landscapes, and execute targeted diagnostic and therapeutic interventions at the subcellular level (Wang et al., 2015) (Table 2).

**TABLE 2:** Summary of barrier-traversal strategies in CHF nanodelivery.

Barrier level	Targeting strategy	Representative nanocarrier	Key design/Mechanism	References
MPS clearance	PEGylation	PEG2000-liposomes	Steric hindrance; prolonged circulation	Sheng et al. (2009), Kanamala et al. (2018), Mustafa et al. (2017)
	Biomimetic coating	PM&EM NPs	“Self” recognition (CD47); integrin αIIbβ3/P-selectin targeting collagen/myofibroblasts	Lu et al. (2023), Li et al. (2022), Cui et al. (2024), Saunders et al. (2020), Sakurai et al. (2002), Li et al. (2024a)
Hemorheology	Size/morphology optimization	Flexible nanogels; ellipsoidal particles	100–200 nm; enhanced margination; EPR in ischemic myocardium	Longmire et al. (2008), Kamal et al. (2025), Lundy et al. (2016), Müller et al. (2014), Blanco et al. (2015)
Endothelium	Active targeting	CTP-modified carriers	Binding to stress fiber proteins/ischemia-induced receptors	Sun et al. (2023), Guan et al. (2022), Wang et al. (2018b), Kanki et al. (2011)
	Contraction-driven extravasation	Ultrasmall CaP	“Squeeze-and-release” via cardiac cyclic stress	Miragoli et al. (2018)
Endosome/lysoso-me	Membrane fusion	MITO-Porter (R8-liposome)	Direct fusion with mitochondrial membrane; bypass lysosomes	Buchke et al. (2022)
	Charge reversal	Ionizable LNPs	pH-triggered membrane fusion; ΔΨ-driven mitochondrial accumulation	Gil-Cabrerizo et al. (2024), Soroudi et al. (2024)

## 6 AI-driven mitochondria-targeted nanomedicine

Mitochondrial dysfunction represents the core pathological driver of CHF progression and constitutes an actionable therapeutic target that determines disease prognosis. Nanomedicine addresses this challenge through the construction of programmable delivery systems capable of traversing hierarchical barriers—from systemic circulation across cell membrane, endosomal/lysosomal compartments, and ultimately the mitochondrial double membrane—to achieve precise enrichment within the mitochondrial matrix. Concurrently, AI enables rational optimization of carrier composition, morphological architecture, and surface functionalization by decoding high-dimensional nano-bio interface data, providing a programmable nanoplatform for reversing myocardial bioenergetic failure. The convergence of these three paradigms not only facilitates the therapeutic transition from hemodynamic compensation to etiological repair but also furnishes novel strategies for precise, efficient, and individualized mitochondria-targeted interventions in CHF.

### 6.1 Machine learning-guided design of antioxidant nanocarriers

The fundamental challenge in achieving targeted drug delivery via nanosystems lies in overcoming biological barriers while enhancing specificity toward particular tissues or cells. Addressing the oxidative stress characteristics of CHF, AI has been instrumental in optimizing nanoformulations of various mitochondria-targeted antioxidants. Coenzyme Q10 (CoQ10), a critical component of the mitochondrial electron transport chain that serves a pivotal role in electron transfer from Complex I/II to Complex III (Parker et al., 2025), has demonstrated cardioprotective effects across multiple preclinical HF models (Awad et al., 2022; De Blasio et al., 2015). The randomized, double-blind Q-SYMBIO trial demonstrated that CoQ10 administration for 106 days significantly reduced major adverse cardiovascular events in HF patients (Mortensen et al., 2014). However, the therapeutic efficacy of CoQ10 is constrained by its poor aqueous solubility and low mitochondrial accumulation efficiency (Wang et al., 2024; Hibino et al., 2019). Machine learning algorithms, by analyzing relationships between molecular descriptors and mitochondrial matrix enrichment coefficients, can optimize surface modifications of CoQ10 nanocrystals to enhance cardiomyocyte uptake efficiency.For more potent mitochondria-targeted antioxidants, 10-(6′-plastoquinonyl) decyltriphenylphosphonium exhibits distinctive advantages (Forini et al., 2020). SkQ1 not only accumulates via membrane potential-driven partitioning but also binds to cardiolipin within the inner mitochondrial membrane, inhibiting the hydrophobic interaction between cardiolipin and cytochrome c, thereby attenuating the peroxidase activity of cytochrome c (Firsov et al., 2016). AI-assisted molecular dynamics simulations enable optimization of lipid membrane composition and particle size distribution in nanoliposomes, enhancing the mitochondria-targeted delivery efficiency of SkQ1. In ischemia-reperfusion injury models, SkQ1 nanoformulations have demonstrated potential in mitigating oxidative stress and preserving myocardial integrity (Burska et al., 2026).

### 6.2 Biomimetic nanoparticles and membrane coating technology

Biomimetic nanoparticles, such as macrophage membrane-coated and platelet membrane-coated nanoparticles, enhance targeted drug delivery by mimicking natural cellular properties (Wang et al., 2021; Yin et al., 2022). In CHF, the accumulation of peroxides resulting from decreased endogenous superoxide dismutase levels can be addressed by designing platelet membrane-coated Tempol nanoparticles under AI guidance. Based on the established pharmacological effects of Tempol in improving left ventricular ejection fraction and antioxidant enzyme activity in PPARα-knockout mice (Guellich et al., 2013), AI provides a proof-of-concept strategy for translating this compound into nanoformulations—specifically, by engineering platelet membrane-coated Tempol nanoparticles and utilizing deep learning to optimize P-selectin density on the membrane surface, theoretically enhancing cardiac targeting efficiency. This computational design approach offers new possibilities for clinical translation of Tempol, though its actual therapeutic efficacy awaits further validation in HF animal models.

Nanotechnology enables the fabrication of diverse mitochondria-targeted drug carriers, including inorganic nanomaterials (e.g., mesoporous silica, alumina nanorods), liposomes, nanomicelles, polymers, and supramolecular assemblies, all of which can be functionalized with mitochondria-targeting moieties to load mitochondrial function-modulating drugs (Wen et al., 2016; Khan et al., 2022). AI-driven molecular dynamics simulations can predict the vascular behavior of nanocarriers with varying physicochemical parameters, theoretically optimizing the mitochondria-targeted delivery efficiency of triphenylphosphonium-modified nanoparticles—a strategy that remains to be experimentally validated.

### 6.3 Mitochondrial transplantation and artificial Mitochondria

Mitochondrial transfer represents a pivotal therapeutic modality for replenishing cellular energy in patients with ischemic heart disease; however, the invasive nature of transplantation procedures and the diminished functionality of transferred mitochondria have constrained its clinical advancement (Buratto and Konstantinov, 2021). Wan et al. developed an oral CM/NM/Mito@Cap capsule that achieves non-invasive mitochondrial transplantation through nanotechnology at the proof-of-concept stage: nanomotors releasing nitric oxide are modified onto the mitochondrial surface, while cardiac membrane fragments are asymmetrically inserted into the surface, all loaded within pH-responsive enteric capsules (Wu et al., 2024). Building upon the bionic principles of this prototype system, AI-enhanced design strategies theoretically enable further optimization of cardiac membrane component density and nanomotor/mitochondria ratios. Through machine learning predictions of optimal parameter combinations for intestinal epithelial cell uptake efficiency and cardiac tropism, this approach theoretically enhances the homing efficiency and fusion functionality of mitochondria within injured myocardium, providing a preclinical foundation rather than a clinically validated platform for the clinical translation of this technology.

To circumvent challenges associated with mitochondrial isolation fragility, purification complexity, and immunocompatibility, the construction of stable artificial ATP synthesis systems independent of external energy represents a feasible alternative strategy. Wan et al. designed oral artificial mitochondria nanorobots with AI-optimized spatial arrangement of functional modules as an early-stage conceptual prototype: a PCr segment that reacts with cytoplasmic ADP to generate ATP, a perfluoroalkyl component that penetrates the intestinal mucus barrier, and an L-arginine segment that targets damaged mitochondria. Upon reaching the injured heart, AMNs rapidly produce ATP to alleviate mitochondrial burden and restore cellular viability in proof-of-concept small-animal settings, providing a theoretical therapeutic avenue for treating mitochondrial dysfunction through *in vivo* energy transfer (Li et al., 2025).

Technology readiness and translational constraints. While the aforementioned mitochondrial transplantation and artificial mitochondria strategies represent conceptually groundbreaking frontiers, it is essential to explicitly acknowledge their current technology readiness levels (TRLs) and the formidable translational obstacles that must be overcome prior to clinical application. The oral CM/NM/Mito@Cap platform (Wu et al., 2024) and artificial mitochondria nanorobots (Li et al., 2025) currently reside at TRL 2–3; neither has advanced to large-animal validation, Good Laboratory Practice toxicology profiling, or regulatory pre-IND consultation. Several critical bottlenecks warrant rigorous scrutiny. First, *in vivo* safety and immunocompatibility: the immunogenic potential of allogeneic or xenogeneic mitochondrial components, the risk of off-target accumulation of synthetic nanomotors and membrane modules in non-cardiac tissues, and the long-term metabolic fate of perfluoroalkyl and phosphocreatine segments remain incompletely characterized. Second, long-term stability and functional durability: the capacity of transplanted mitochondria or artificial ATP-generating systems to maintain structural and bioenergetic integrity within the chronically oxidative, protease-enriched, and calcium-dysregulated microenvironment of the failing myocardium is uncertain; moreover, the therapeutic durability beyond acute delivery windows and the safety implications of repeated dosing—particularly regarding cumulative nanomaterial toxicity and immunological priming—are entirely unexplored. Third, scalable manufacturing and quality control: current protocols for mitochondrial isolation, purity assessment, membrane functionalization, and nanorobot module assembly are labor-intensive, poorly standardized, and subject to substantial batch-to-batch variability, presenting profound challenges to Good Manufacturing Practice compliance, analytical validation, and cost-effectiveness at clinical scale. These limitations do not negate the visionary innovation underlying these platforms but underscore the imperative that AI-driven design optimization must proceed in parallel with rigorous preclinical toxicology, scalable bioprocess engineering, and adaptive regulatory science to bridge the chasm between bench-top prototypes and clinically viable therapeutics (Table 3).

**TABLE 3:** AI-driven mitochondria-targeted nanotherapeutics in CHF.

Therapeutic modality	AI-empowered design	Representative nanocarrier	Preclinical outcome	References
Antioxidant nanoformulatio-ns	ML-optimized surface/MD simulation	CoQ10 nanocrystals/SkQ1 liposomes	Enhanced mitochondrial uptake; reduced oxidative stress	Parker et al. (2025), Awad et al. (2022), De Blasio et al. (2015), Mortensen et al. (2014), Wang et al. (2024), Hibino et al. (2019), Forini et al. (2020), Firsov et al. (2016), Burska et al. (2026)
Biomimetic nanoparticles	DL-optimized membrane density/MD prediction	Platelet membrane–coated Tempol NPs/TPP^+^-modified carriers	Improved LVEF/antioxidant activity (Tempol); ΔΨ-driven matrix enrichment	Wang et al. (2021), Yin et al. (2022), Guellich et al. (2013), Wen et al. (2016), Khan et al. (2022)
Mitochondrial transplantation and artificial organelles	ML-predicted optimal component ratios	CM/NM/Mito@Cap/AMNs	Non-invasive mitochondrial delivery; *in vivo* ATP replenishment	Wu et al. (2024), Li et al. (2025)

## 7 Discussion

This Review systematically delineates the innovative strategies and translational prospects arising from the convergence of artificial intelligence and nanotechnology in mitochondria-targeted therapy for CHF. Anchored in the mechanistic foundation that mitochondrial dysfunction serves as the core pathological driver of CHF progression, we have comprehensively examined how nanocarriers, through precision engineering, transcend the off-target limitations of conventional pharmacological agents. By overcoming hierarchical physiological barriers—from the cardiac microvascular endothelium to the mitochondrial double membrane—these systems achieve subcellular cascade-targeted delivery with unprecedented spatial resolution. Critically, AI technologies now permeate the entire lifecycle of nanomedicine development: from machine learning optimization of lipid nanoparticle endosomal escape efficiency, to molecular dynamics simulations dissecting the microscopic kinetics of protein corona formation, and further to big data-driven intelligent screening of mitochondria-affine ligands. This AI-enabled rational design has not only significantly enhanced the bioavailability of antioxidants such as coenzyme Q10 and SkQ1, but has also propelled revolutionary therapeutic modalities—including mitochondrial transplantation and artificial mitochondria nanorobots—from proof-of-concept validation toward preclinical application.Such technological convergence heralds a paradigm shift in CHF therapeutics: the field is transitioning from traditional hemodynamic compensation management toward a new era of “etiological repair” predicated on mitochondrial bioenergetic remodeling.

Despite the tremendous promise of the AI-nanomedicine convergence in mitochondria-targeted interventions, substantial multidimensional limitations persist in achieving genuine clinical translation. Mitochondrial transplantation microcarriers and artificial mitochondria nanorobots, while conceptually transformative, remain at low technology readiness levels and face substantial translational hurdles encompassing *in vivo* immunosafety. Currently, AI model training is critically dependent on high-quality nano-bio interaction datasets. However, existing nanomedicine databases often suffer from data fragmentation, insufficient standardization, and limited sample sizes; high-dimensional datasets specifically dedicated to mitochondria-targeted are particularly scarce. Consequently, AI models face risks of overfitting and poor generalization when predicting nanocarrier behavior in complex pathological environments, creating a pronounced translational gap between *in vitro* predictions and *in vivo* efficacy. More fundamentally, the dynamic fission/fusion mechanisms and heterogeneity of mitochondria across different CHF stages remain incompletely elucidated. Although AI can optimize carrier design, even the most precise delivery systems cannot yield anticipated therapeutic benefits if the biological targets themselves are misidentified or therapeutically inappropriate.

Mitochondria-targeted antioxidants including SkQ1, CoQ10, and MitoQ demonstrate significant cardioprotective effects in acute myocardial injury models; however, safety data regarding chronic intervention remain largely absent, particularly concerning the potential “re-oxidation” risk. Upon drug discontinuation or acute stress exposure, downregulated endogenous antioxidant defense systems may prove incapable of responding to sudden oxidative stress bursts, potentially exacerbating cardiomyocyte injury. Furthermore, AI-optimized nanoformulations, through enhanced mitochondrial matrix accumulation, may aggravate the risk of mitochondrial calcium overload. If antioxidant delivery via nanocarriers is not coordinated with calcium homeostasis regulation, the improvement of oxidative stress may occur concurrently with disruption of mitochondrial calcium buffering capacity, triggering mPTP opening and cell death cascades. Currently, longitudinal toxicological and pharmacokinetic monitoring systems specifically designed for AI-engineered nanomedicines remain undeveloped, with algorithmic models for predicting myocardial accumulation of nanomaterials and the toxicity of metabolic byproducts lacking—factors that substantially constrain clinical translation.

The construction of patient-specific mitochondrial damage atlases through integration of genomics, proteomics, metabolomics, and radiomics data necessitates prohibitively high detection costs. Compounded by the fact that CHF patients are predominantly elderly with multiple comorbidities and already substantial healthcare burdens, this creates a direct implementation dilemma arising from the tension between technological sophistication and healthcare accessibility. If mitochondria-targeted therapies optimized by AI can only benefit patients in developed regions with comprehensive omics detection resources, such disparities will exacerbate global health inequities.

Sexual dimorphism manifests significantly across human diseases, including cardiovascular disorders (Shi et al., 2024). Males face a higher lifetime risk of heart failure with reduced ejection fraction, whereas females predominantly develop heart failure with preserved ejection fraction (van Essen et al., 2025); furthermore, US data indicate that heart failure mortality is higher among women across all age groups (Virani et al., 2021). These disparities arise not only from sex-specific neurohormonal and bioenergetic remodeling (Pereyra et al., 2026) but are also profoundly shaped by epigenetic regulation. Specifically, lifestyle factors and environmental exposures can induce epigenetic modifications—including DNA methylation, histone alterations, and non-coding RNA reprogramming—via estrogen-related compounds, with these changes transmissible through germlines, thereby further molding cardiovascular susceptibility and mitochondrial phenotypic variability (Shi et al., 2024). These biological disparities extend directly into the nanotherapeutic domain. Sex significantly modulates nanoparticle pharmacokinetics, biodistribution, and immune-mediated clearance. In animal experiments, females typically exhibit slower mononuclear phagocyte system (MPS)-mediated elimination, higher renal accumulation, and distinct protein corona profiles. Of particular importance, a bidirectional regulatory axis exists between sex hormones and nanocarriers: nanoparticles can perturb sex hormone secretion, while estrogen and testosterone conversely alter nanoparticle absorption and endosomal processing (Poley et al., 2022). Recent investigations employing sex-stratified cardiac organoids have revealed marked dimorphisms in transcriptomic signatures (e.g., MEG3, WDR72), sarcomeric ultrastructure, and tissue-scale biomechanical properties; nevertheless, under baseline conditions, nanoparticle distribution showed no significant sex-based divergence, suggesting that the absence of immune components in current models may obscure sex-specific nano-bio interactions that would otherwise emerge in a more physiologically complete microenvironment (Grumelot et al., 2026). Given that the majority of preclinical nanomedicine studies continue to rely exclusively on male mice, this methodological bias risks systematically concealing sex-specific therapeutic responses, off-target toxicities, and mitochondrial bioenergetic profiles (Poley et al., 2022). Therefore, amid growing emphasis on precision medicine and personalized drug delivery, the convergence of artificial intelligence and nanomedicine must treat sex as a non-negotiable stratification variable—constructing sex-disaggregated, multi-omics-derived mitochondrial damage atlases and rigorously auditing machine learning datasets for sex representation bias—to ensure that therapeutic optimization reflects the full spectrum of patient biology.

To overcome the above bottlenecks and advance the field beyond the proof-of-concept stage. First, self-driving AI laboratories integrated with standardized data factories will directly remedy the data infrastructure deficits that currently constrain model generalizability. By combining robotic automation, microfluidic high-throughput synthesis, and closed-loop machine learning feedback, these platforms can generate large-scale, standardized nano-mitochondrial interaction datasets and achieve real-time iterative training. Second, patient-specific digital twin heart models will provide critical *in silico* tools to bridge the in vitro–in vivo translational gap. For CHF patients characterized by advanced age, multiple comorbidities, and marked sex differences, static multi-omics atlases are insufficient to achieve dynamic precision matching. Future research should deeply integrate genomic, proteomic, metabolomic, and radiomic data to reconstruct the spatiotemporal evolution trajectories of individual mitochondrial damage. Leveraging deep learning-driven physiologically-based pharmacokinetic simulation, digital twin platforms can, prior to administration, simulate *in silico* the cascade penetration of nanocarriers across cardiovascular barriers, subcellular distribution, and mitochondrial functional recovery kinetics in specific patient avatars. This approach can prospectively identify off-target toxicity, predict sex-specific nano-bio interface responses, and reduce R&D costs through virtual clinical trials, thereby alleviating the disparity between technological sophistication and global healthcare accessibility. Moreover, such platforms may integrate Chinese and Western medicine co-intervention modules, providing mechanistic dissection and dose optimization for the combined application of traditional Chinese medicine formulae with AI-engineered nanocarriers.Finally, even with AI-optimized design and digital twin pre-validation, frontier modalities such as mitochondrial transplantation microcarriers and artificial mitochondria nanorobots remain at low technology readiness levels, facing unresolved challenges in scalable manufacturing, long-term toxicological profiling, and adaptive regulatory pathways. Concurrent advancement of scalable bioprocess engineering compliant with Good Manufacturing Practice standards, adaptive regulatory science frameworks, and longitudinal pharmacokinetic-toxicological monitoring algorithms specifically designed for AI-engineered nanomedicines is essential to establish the complete translational chain from computational design, digital twin preclinical rehearsal, automated manufacturing, and ultimately clinical validation, thereby realizing the paradigm shift from hemodynamic compensation to mitochondrial etiological repair.

## 8 Conclusion

The occurrence and progression of chronic heart failure are closely associated with persistent mitochondrial dysfunction, mainly manifested as impaired energy metabolism, excessive oxidative stress, calcium homeostasis imbalance, and activation of inflammatory and apoptotic pathways. Mitochondrial abnormalities are not only important drivers of disease progression but also provide key therapeutic targets for intervention. Mitochondria-targeted nanocarriers demonstrate significant advantages in improving drug stability, enhancing tissue and subcellular targeting, and traversing multiple biological barriers, offering novel insights for precision therapy in chronic heart failure. Meanwhile, the introduction of artificial intelligence provides new technical support for the design and optimization of nanomedicines. Through its application in targeted ligand screening, carrier structure design, drug release prediction, and personalized dosing regimen formulation, artificial intelligence is expected to further enhance the efficiency and precision of mitochondria-targeted delivery systems. However, mitochondria-targeted nanotherapy still faces numerous challenges before achieving broad clinical translation; future efforts must strengthen multidisciplinary collaboration, promote deep integration of experimental research and computational methods, and facilitate the coordinated development of precision nanomedicine and clinical cardiovascular therapeutics.
